# Supplementary material for: Neuroreceptor Activation by Vibration-Assisted Tunneling
Source: Sci Rep. 2015 Apr 24;5:9990. doi: 10.1038/srep09990 (PMC4408984; doi:10.1038/srep09990)
Supplement: Supplementary Information [file srep09990-s1.pdf]

Neuroreceptor Activation by Vibration-Assisted Tunneling : Supplemental Material

Ross D. Hoehn,[1] David Nichols,[2] Hartmut Neven,[3] and Sabre Kais[4,5]

[1] *Department of Chemistry, Purdue University, West Lafayette, IN 47907 USA*

[2] *Department of Medicinal Chemistry and Molecular Pharmacology, Purdue University, West Lafayette, IN 47907 USA*

[3] *Google, Venice, CA 90291 USA*

[4] *Departments of Chemistry and Physics, Purdue University, West Lafayette, IN 47907 USA*

[5] *Qatar Environment and Energy Research Institute, Qatar Foundation, Doha, Qatar*

I. TUNNELING THEORY

Inelastic Electron Tunneling Spectroscopy is a well-founded experimental method utilizing a simplistic laboratory set-up that can deliver the vibrational spectra of an analyte. The mechanism of action is semi-classical and not optical, particular selection rules are derivable with IETS[1] but in general this method allows for forbidden transitions, thus all vibrational modes are addressable[2]. The method is implemented by the application of a potential across a two-plate junction with a spatial separation between the plates. High energy electrons from the valence band of one plate will tunnel across the junction into the conduction band of the other. When the tunneling process occurs in the absence of analyte molecules, the process is elastic in nature and electron energy is maintained throughout the process, thus the electrons energy must be respective of the energy between the valence and conductance band.

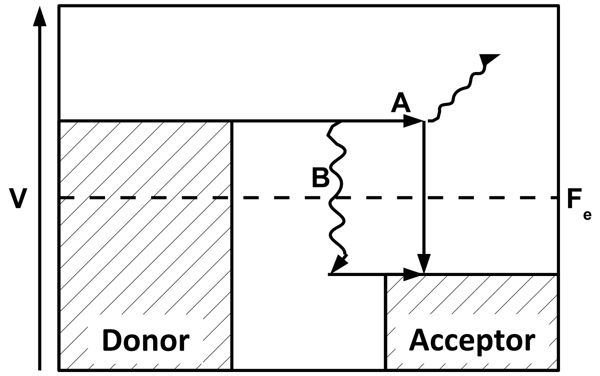

FIG. 1: Cartoon displaying the competing processes during IETS where V is potential energy and F_e is the Fermi Level. Path A is radiative[1,3], requiring the tunneling electron to spontaneously lose energy to meet the energy of the conductive band. Path B shows the electron losing energy via a non-radiative process; it is implicit that the energy lost is to a normal mode of a deposited molecule within the gap - such is our case.

Depositing an analyte molecule onto the electron source plate, as the tunneling electrons enter the gap they may interact with the deposited analyte molecules; in doing so they are effectively given a springboard, shortening their tunneling path. This interaction comes at a cost of energy; the electron will lose energy to the analyte molecule, where the amount of lost energy is equal to that of a vibrational mode of the molecule. This process may be seen in Figure 1. This method has been well described theoretically[4–6] and expanded to include such considerations as molecular orientation[7] and short-ranged higher harmonics[8]. Here we shall review the theoretical description of the elastic process a seen in[9,10]. It is a fair starting assumption that the wave function is oscillatory in the x- and y-directions and evanescent in the z-direction. In this manner the decay constant for such a function is spatially dependent and thus the function is anisotropic; the wave functions used were described

through WKB theory and are provided here:

$$\begin{cases} \psi_i = \left(\frac{A}{L}\right) e^{i\mathbf{k}'_{||}\cdot\mathbf{r}} e^{-\alpha_0 z} \\ \psi_f = \left(\frac{A}{L}\right) e^{i\mathbf{k}_{||}\cdot\mathbf{r}} e^{-\alpha_0 (d-z)} \end{cases} \quad (1)$$

From the above it should be noted that L is the dimension of the square plate. k_x, k_y and k_z are the wave vectors in the appropriate directions. Similarly, A is a collection of constants, α_0 is the decay rate of the evanescent wave in the z direction and assuming cylindrical symmetry the wave vectors in the $\hat{x}$ and $\hat{y}$ directions are identical and are combined into the radial wave vector $k_{||}$, noting that this is the wave vector components parallel to the plate surface.

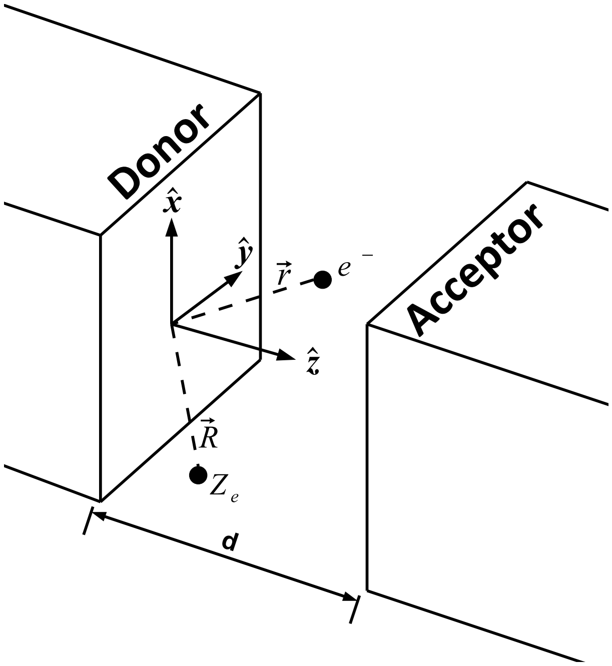

FIG. 2: A cartoon displaying the physical relations and significance of variables within the problem. e^- is the tunneling electron with vector displacement of $\vec{r}$ and Z_e is the partial charge associated with a molecular mode with displacement $\vec{R}$, and d is the distance between the two plates.

We shall use the wave functions in Eq. 1 to attain the average value of the current for the system, via the current operator, M_e. The elastic process yields:

$$< \psi_i|\hat{M}_e|\psi_f >= M_e = \left(\frac{A^2}{L^2}\right) \int\limits_0^L dS e^{i\mathbf{q}\cdot\mathbf{r}} \left(\frac{\hbar^2\alpha_0}{m}\right) e^{-\alpha_0 d} \tag{2}$$

$$= \left(\frac{A^2\hbar^2\alpha_0}{m}\right) \cdot e^{-\alpha_0 d} \ , \tag{3}$$

where $\mathbf{q}$ is the difference between $\mathbf{k}'_{||}$ and $\mathbf{k}_{||}$. The inelastic process for a single specified normal mode is governed by the following interaction potential:

$$U(r') = \frac{Z_e e^2}{4\pi\epsilon_0\epsilon_r} \cdot \frac{\mathbf{u}\cdot(\mathbf{R}-\mathbf{r})}{(|\mathbf{R}-\mathbf{r}|)^3} \ , \tag{4}$$

where all symbols retain their standard definitions, including ϵ_r being the permittivity of the generic real media, $\mathbf{r}$ and $\mathbf{R}$ are made clear by Figure 2 and $\mathbf{u}$ is the vector representing the displacement of the atom within the molecule with partial charge Z_e. This potential allows us to calculate the inelastic contributions in a manner similarly to the above:

$$M_{in} = \left(\frac{A^2}{L^2}\right) e^{-\alpha_0 d} e^{i\mathbf{q}\cdot\mathbf{R}} \int_0^\infty \int_0^{2\pi} \int_0^d r e^{i\mathbf{q}\cdot\mathbf{r}} U(r,\theta,z) dz d\theta dr \ . \tag{5}$$

Where the integral in Equation 5 can be performed analytically for cases where the vector directions of $\mathbf{u}$ are either parallel or perpendicular to the plate surfaces. For $\mathbf{u}$ along the z direction (parallel to gap):

$$M_{in}^z = M_0 \frac{1}{qd}\left(e^{-q\alpha_0} - e^{-q(d-\alpha_0)}\right) \ ; \tag{6}$$

and for $\mathbf{u}$ parallel to the plates:

$$M_{in}^x = iM_0 \frac{1}{qd}\left\{\left(1 - e^{-q\alpha_0}\right) + \left(1 - e^{-q(d-\alpha_0)}\right)\right\} \ . \tag{7}$$

Where, in both the above, the quantity M_0 is given by:

$$M_0 = e^{i\mathbf{q}\cdot\mathbf{R} - \alpha_0 d} \left(\frac{A^2 Z e^2 u d}{L^2 2\epsilon_0\epsilon_r}\right) \ . \tag{8}$$

The decay constants for each of ψ_i and ψ_f should conform with the statement:

$$E_c - E = \frac{\hbar^2}{2m}(\alpha^2 - k_{||}^2) \tag{9}$$

where E is the energy of the tunneling electron, E_c is the energy of the conductive band and m is the mass of the electron (effective mass is typically used). The above yields two unique decay constants consistent with the difference in electron energies at the conduction band and during tunneling. With these two unique decay constants we must append a factor of

$$e^{(\alpha_i+\alpha_f)d/2} e^{(\alpha_i-\alpha_f)z} \tag{10}$$

to our matrix elements due to the difference in α's. Carrying this factor through we note there is a depletion of tunneling probability equivalent to:

$$e^{-q^2/4(\alpha_0 d)}; \tag{11}$$

and finally placing this into an expression for the relative conductivities associated with the inelastic and elastic processes, $\frac{\Delta\sigma}{\sigma_e}$, and finally including a 2-D density of states representative of the plate surface areas:

$$\frac{\sigma_{in}}{\sigma_e} = \left\{\frac{1}{M_e}\right\}^2 \int_0^\infty (M_{in}^z)^2 \, e^{-q^2/4(\alpha_0/d)} \left(\frac{qL^2}{2\pi}\right) dq. \tag{12}$$

The above allows us to make the statement:

$$\frac{\sigma_{in}}{\sigma_e} \propto Z_e^2 u_z^2, \tag{13}$$

as those quantities on the R.H.S. of Eq. 12 are the only quantities dependent on molecular characteristics and thus are featured in Eq. 13. As the elastic tunneling process occurs with or without the presence of the analyte molecules, the experimental observable is the ratio between the known elastic contribution, $\sigma_e = M_e^2$, to the current at a given applied potential (found through a zeroing process with a

non-deposited gap) and the deposited gap current at the same potential; this ratio quantity is denoted as $\frac{\Delta\sigma}{\sigma_e}$. Armed with the above, the IETS intensity for a given active mode j can be approximated by[6,11]:

$$I_j = \sum_{i=1}^{N} I_{i,j} = \sum_{i=1}^{N} q_i^2 (\Delta x_{i,j})^2, \tag{14}$$

where the sum is over all atoms within the molecule, q_i is the partial charge of atom i, and $\Delta x_{i,j}$ is the Cartesian displacement of atom i in mode j.

II. DISCUSSION OF TUNNELING SPECTRAL ASPECTS

An examination of the edrogenous agonist 5-HT is given in Figure 3. The main spectral features are (quantities are in cm^{-1}): the OH stretch at 3700; NH_2 bend at 1700; coherent ring motions appear at both 1500 and 1150; and indole bending at 530. For reasons discussed below, we will focus our discussion on tunneling in the 1500 cm^{-1} region. Working within Turins theory, this implies that these motions assist in the turnneling and that the tunneling source and sink are in proximity to these motions. Docking studies of homology modeled 5-HT_{2A} show that the moieties discussed above are local to F339, F340, S159 and L229 residues[12–15], alluding that one of these residues may facilitate the tunneling process.

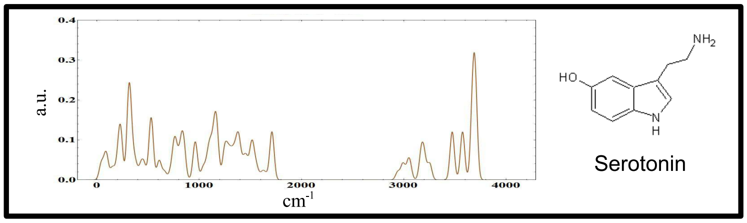

FIG. 3: Serotonin's tunneling spectrum used as a comparitor throughout the discussion. Here the abscissa has units of wavenumber and the ordinate has units proportional to tunneling probability; this convension holds for all tunneling spectrum to follow.

The spectral indices are given for the overall spectrum and followed by regional SI's calculated for spans of 1000cm^{-1} with 500cm^{-1} steps, to emphasize the possible region associated with acitvation the final column shows the SI of each compound for 1500$\pm$100cm^{-1}. Regions with large ranges of zero intensity have SI's inflated by this spectral facet, these regions have been disincluded within the table. When disincluding these regions, the SI for the region spanning 1000-2000cm^{-1} shows enhanced values, and includes the peak at 1500cm^{-1}. The final column of the table gives the SI for a 100cm^{-1} region about this peak to emphasis this heavily shared spectral feature. Application of the SI to the square of the spectra yielded similar results (not shown), yet with the expected enhancement of the SI values (not shown). These values were compared to experimental EC50 values, representative of pharmacological potency, taken from the work of Parrish and Braden[16]. These values have been tabulated and are collect within Table II

In the next few sections we have selected DOC (2,5-dimethoxy-4-C-amphetamines) as a prototypical molecule for discussion, this selection was based on its fairly tractable number of modes, simple geometry, symmetry and similarities to other agonists. Energy regions associated with an assisted electron transfer would bene-

	Spectral Range (cm^{-1})					
	0-4200	0-1000	500-1500	1000-2000	3000-4000	1400-1600
LSD	1	1	1	1	1	1
DAM-57	0.89019	0.87090	0.85286	0.87048	0.86997	0.85726
2C-I	0.81664	0.81792	0.75492	0.76284	0.78236	0.70693
2C-T-7	0.83977	0.81371	0.77398	0.80550	0.81748	0.79913
DOI	0.81903	0.80415	0.76196	0.77684	0.78614	0.71396
Aleph-2	0.83233	0.82218	0.77757	0.79102	0.80063	0.77539
DMT	0.84620	0.82282	0.78969	0.80327	0.85075	0.75394
Mescaline	0.82280	0.80003	0.74899	0.77347	0.80797	0.76743
Quipazine	0.82353	0.80820	0.77809	0.79677	0.77694	0.72653
Benzylpiperazine	0.82135	0.79383	0.76929	0.79990	0.79270	0.72390

TABLE I: Table contains the SI indexes for several 5-HT$_{2A}$ agonists. The procedure was applied to the total spectra, and several sections of $1000cm^{-1}$ which march with an overlaping pattern and shifted by 500cm^{-1}. The region of interest is also performed with a calculated SI for the region of $1500\pm100^{cm-1}$

fit from a large density of vibrational states; implying a greater number of possible states to interact with in this energy range. Figure 4 shows both the IETS and scaled density of states for DOC; the spectral feature at 1500cm^{-1} exhibits an enhanced number of vibrational states.

	h5-HT_{2A}	A20 5-HT_{2A}	
Drugs	K_i [^{125}I]DOI (nm)	EC_{50} (nm)	Intrinsic Activity (% 5-HT)
(±)-DOB	0.83(0.18)	17.4(3.0)	70.2(4.0)
2C-B	1.13(0.13)	18.2(2.3)	40.0(4.5)
(±)-DOI	0.60(0.04)	9.7(1.8)	49.7(2.2)
2C-I	0.70(0.05)	9.8(1.7)	30.3(1.5)
(±)-DOTFM	0.46(0.02)	10.1(1.6)	50.1(3.7)
2C-TFM	0.63(0.07)	7.7(1.5)	25.7(1.6)
(±)-Aleph-2	0.70(0.05)	13.1(1.6)	57.0(4.4)
2C-T2	0.78(0.02)	14.4(2.1)	43.5(4.6)

TABLE II: Table contains EC50 data used for comparison to our calculated results of electron tunneling. Data is from Parrish and Braden[16].

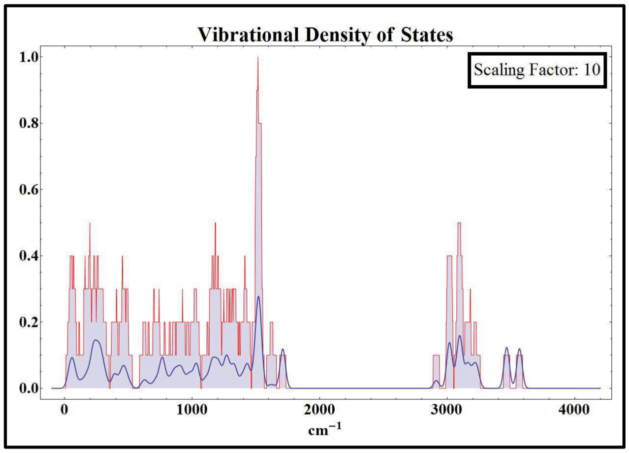

FIG. 4: The tunneling spectrum of DOC (Blue) is plotting alongside a scaled, discrete density of states for the vibrational modes of DOC. The scaling factor is given in the inlay. Note the enhanced number of states associated with 1500cm^{-1} region. This large number of states reflects a large density of possible vibrational modes within this energy range that are capable of accepting a quanta of energy from the tunneling electron.

In the main body of the paper we propose an isotopolgue series for DAM-57; the series is of variants are dueterated functional groups altering the character in the 1500cm^{-1} region. We verified that isotopologues of other atoms do not to alter tunneling character in this region. Figure 5 shows the isotope effects within several groups of the molecule. Fig. 5 a) shows the effects of replacing the oxygens with ^{18}O's, this results in little alteration near 1500cm^{-1}; substitution of the halide has similar results, with differences appearing at much lower energies. Fig. 5 b) displays the effects of dueterating the hydrogens on the methoxys, this show a large attenuation of the tunneling intensity; finally, Fig. 5 c) shows the effect of selectively dueterating different functional groups.

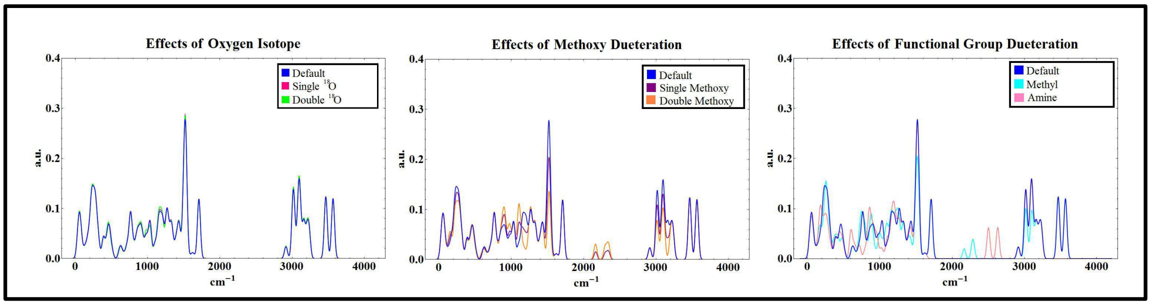

FIG. 5: Plots of the isotoplogues of oxygen with the DOC molecules. The isotope exchanges has no effect on the region in question. This means that it is not vibration of the oxygen atoms which are most heavily responsible for the tunneling features within our candidate region.

BIBLIOGRAPHY

J. Lambe and R. C. Jaklevic, Phys. Rev. **165**, 821 (1968).

K. W. Hipps and U. Mazur, *Inelastic Electron Tunneling Spectroscopy*, Handbook of Vibrational Spectroscopy, Wiley (2001).

J. Lambe and S. L. McCarthy, Phys. Rev. Lett. **37**, 923 (1976).

A. K. Sleigh, M. E. Taylor, C. J. Adkins and W. A. Phillips, Journal of Physics: Condensed Matter **1**, 1107 (1989).

C. J. Adkins and W. A. Phillips, Journal of Physics C: Solid State Physics **18**, 1313 (1985).

A. K. Sleigh, W. A. Phillips, C. J. Adkins and M. E. Taylor, Journal of Physics C: Solid State Phsyics textbf19, 6645 (1986).

J. Kirtley and J. T. Hall, Phys. Rev. B **22**, 848 (1980).

J. Kirtley and P. Soven, Phys. Rev. B **19**, 1812 (1979).

J. Kirtley, D. J. Scalapino and P. K. Hansma, Phys. Rev. B **14**, 3177 (1976).

W. A. Phillips and C. J. Adkins, Philosophical Magazine Part B **52**, 739 (1985).

L. Turin, Journal of Theoretical Biology **216**, 367 (2002).

M. R. Braden and D. E. Nichols, Molecular Pharamacology **72**, 1200 (2007).

M. S. Choudhary, N. Scahs, A. Uluer et al., Molecular Pharmacology **47**, 450 (1995).

M. R. Braden, J. C. Parrish, J. C. Naylor and D. E. Nichols, Molecular Pharmacology **70**, 1956 (2006).

J. J. Chambers and D. N. Nichols, Journal of Computer-Aided Molecular Design **16**, 511 (2002).

J. C. Parrish, M. R. Braden, E. Grundy, et al., J Neurochem. **95**, 1575 (2005).
